# Evaluation of Probiotic Growth in Microenvironments Sculpted with Different Geometries

**DOI:** 10.1021/acsomega.4c11168

**Published:** 2025-05-26

**Authors:** Adriano J. G. Otuka, Jonathas Q. R. Moraes, Analú Barros de Oliveira, Eduardo J. S. Fonseca, Carla R. Fontana, Cleber R. Mendonca

**Affiliations:** † Institute of Geosciences and Exact Sciences, Department of Physics, São Paulo State University (UNESP), 13506-900 Rio Claro, SP, Brazil; ‡ São Carlos Institute of Physics, University of São Paulo, CP 369, 13560-970 São Carlos, SP, Brazil; § School of Dentistry, Department of Dental Materials and Prosthodontics, São Paulo State University (UNESP), 14801-903 Araraquara, SP, Brazil; ∥ Grupo de Óptica e Nanoscopia, Instituto de Física, Universidade Federal de Alagoas (UFAL), Campus A.C. Simões, 57072-970 Maceió, AL, Brazil; ⊥ School of Pharmaceutical Sciences, Department of Clinical Analysis, São Paulo State University (UNESP), 14800-903 Araraquara, SP, Brazil

## Abstract

Probiotics benefit their host, potentially exerting microbial balance by stimulating the increase in beneficial bacteria in the intestinal environment. Some studies have shown that specific probiotic strains can alleviate symptoms of medical conditions such as Alzheimer’s, reduce the action of carcinogenic agents, and control various biomarkers in women in the first half of pregnancy. In this context, it is important to determine the fundamental aspects of probiotic growth to develop more efficient delivery mechanisms in pharmaceuticals or foods. Miniaturized biomimetic environments can be useful for that purpose. In this way, we manufactured biocompatible three-dimensional platforms using two-photon absorption polymerization to study the growth of a pool of bacteria composed of Lactobacillus acidophilus, Lactobacillus rhamnosus, Lactobacillus paracasei, and Bifidobacterium lactis, commonly used in commercial probiotics. The microstructures were fabricated using an acrylic resin employing 100 fs pulses from a Ti:sapphire laser. It was possible to manufacture biocompatible structures for probiotic development, demonstrating that microstructures serve as accelerators for bacterial growth. We evaluated the growth of bacteria in the environments over more than 36 h, giving all conditions for their development. Furthermore, it was observed that bacteria grow into structures with distinct geometries (circular or rectilinear) but tend to develop preferentially in protected environments with spacings on the order of 5 μm.

## Graphical abstract

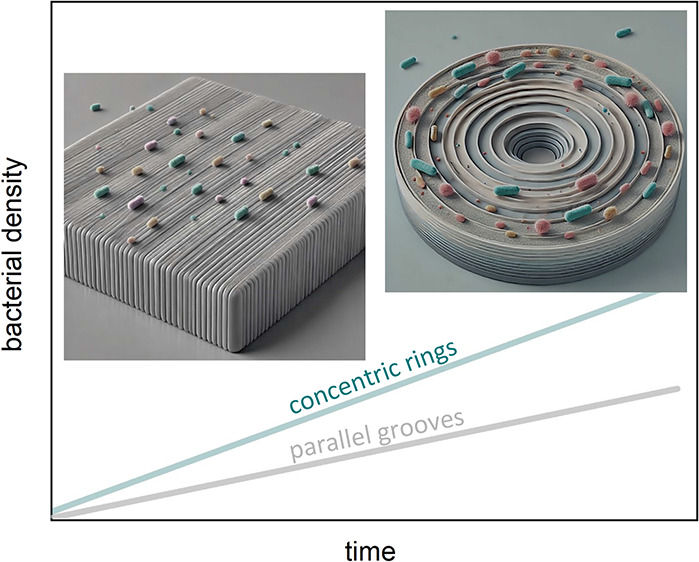

## 1 Introduction

Two-photon absorption polymerization (2PP) has been used to manufacture devices with applications in a plethora of areas of knowledge,^1–4^ having acquired relevance in recent years. Its use has become notable in the production of biomedical microdevices, such as microneedles^5^ and microvalves.^6,7^ In the context of bacterial culture, synthetic matrices have been employed to study the behavior of microorganisms across different geometries and materials with the aim of elucidating the complexity of the signaling mechanisms involved in their development.

In recent years, the 2PP technique has become a valuable tool for this purpose as it allows the fabrication of three-dimensional structures with precise control over size and shape at scales relevant to the study of microorganisms, allowing and obtaining unique and promising results. Biocompatible platforms intended for biological studies have been built using 2PP to investigate the development of eukaryotic and prokaryotic organisms in microenvironments.^8–16^ Additionally, such platforms have been created, for instance, to evaluate the development of nematodes,^17^ bacteria,^18,19^ and cells.^20–22^ In general, two approaches are employed in designing these microenvironments: either aiming to mimic or simulate the characteristics of the biological environment in which the organisms will develop or seeking to understand which geometric aspects influence their development. In either case, the microenvironments must have micrometer-scale dimensions, submicrometric-scale roughness, provide mechanical support, allow for some level of cell confinement (niches), enable optical access for observation, and permit uniform cell distribution and homogeneous nutrient dispersion.^19,21,23–26^

Probiotics are live microorganisms, primarily bacteria and yeasts, that offer health benefits when consumed in adequate amounts. They are often referred to as “good” or “friendly” bacteria because they help maintain the balance of the gut microbiota, which is crucial for overall health.^27^ In this context, one of the most well-known benefits of probiotics is the positive impact on digestive health, resulting in alleviating symptoms of gastrointestinal disorders like irritable bowel syndrome, inflammatory bowel disease, and diarrhea and promoting regular bowel movements and, in consequence, improving digestion.^28^

In addition, probiotics have attracted attention due to positive immunological effects in the host influencing the increase of beneficial bacteria in the intestinal microbiota to the detriment to potentially unwanted microorganisms and consequently reinforcing the host’s equilibrium.^29^ Moreover, probiotic bacteria secrete antibiotics as they prevail in the intestinal flora, inhibiting the action of pathogens in the human digestive tract, a reflection of amensalism and intraspecific competition.^30^

A significant portion of the immune system is located in the gut. Probiotics enhance the body’s immune response by promoting the production of natural antibodies and stimulating immune cells like T-lymphocytes and IgA-producing cells. This helps the body combat infections more effectively and reduces the likelihood of respiratory and urinary tract infections.^28^

Emerging research suggests a strong link between the gut and brain, known as the gut-brain axis. This relationship may help alleviate symptoms of mental health conditions, such as anxiety, depression, and stress, by improving gut health. Some strains of probiotics, like *Lactobacillus* and *Bifidobacterium*, have been shown to positively impact mood and cognitive functions.^30^

Bacteria from the *Lactobacillus* and *Bifidobacterium* genera are probiotics commonly found in the human gut microbiota.^29,31^ These bacteria are exposed to harsh pH environments throughout the human digestive tract, the host’s immune response, and even interspecies competition with other biotic agents. In response to these challenges, it is common for bacteria of a particular species to aggregate into a biofilm rather than remain solitary. A biofilm is a bacterial aggregate that produces an amorphous extracellular matrix composed of proteins, lipids, oligosaccharides, and even DNA, acting as a protective layer that more effectively preserves the community. This form of colonization typically occurs when bacteria adhere to specific types of epithelial cells such as those in the intestinal mucosa.

Various biological mechanisms have been identified as those in which probiotics play a role and may have associated positive effects. Some of these mechanisms include the following: improved digestibility due to the partial degradation of proteins, lipids, and carbohydrates by probiotics; enhanced nutritional value, with higher levels of B-complex vitamins and amino acids such as methionine, lysine, and tryptophan; anticancer action attributed to both the stimulation of the immune system and the breakdown of potentially carcinogenic compounds; and hypocholesterolemic action involving the production of cholesterol synthesis inhibitors.^32^

With a focus on the vast potential for the development of microorganisms in biocompatible microstructures manufactured through 2PP, this work aims to study the behavior of probiotic bacterial development in these environments. More specifically, we used 2PP to fabricate polymeric microenvironments composed of concentric rings (CRs) and parallel grooves (PGs) to study the influence of geometrical features on the growth of a probiotic pool comprising Lactobacillus acidophilus, Lactobacillus rhamnosus, Lactobacillus paracasei, and Bifidobacterium lactis and its dynamics. Also, structures with different spacings between grooves were fabricated to evaluate and corroborate the influence of site dimensions on bacterial growth. We observed significant growth of bacteria in all sculpted geometries, but it was evident in the preferential development of microorganisms in protected environments with spacing on the order of 5 μm.

## 2 Experimental Section

The microstructures, termed microenvironments, were fabricated using a 2PP setup comprising a Ti:sapphire oscillator emitting 100 fs pulses centered at 780 nm with an 86 MHz repetition rate. The laser beam passed through a half-wave plate and a polarizer for intensity adjustment. Then, the beam reflects off a pair of galvanometric mirrors before being focused onto the sample by a 0.25 NA microscope objective. Fabrication is controlled by dedicated software, which governs the positioning of the beam along the *x* and *y* axes via galvanometric mirrors. At the same time, a motorized stage adjusts the *z* position of the sample. The structures were fabricated on a glass substrate using laser pulse energy on the order of 0.8 nJ. Further fabrication specifications can be found elsewhere.^33–36^

The photoresist comprises three monomers, *tris­(2-hydroxy ethyl)­isocyanurate tryacrylate* (SR368 – Sartomer), e*thoxylated (6) trimethylolpropane triacrylate* (SR499 – Sartomer) and *dipentaerythritol pentaacrylate* (SR399 – Sartomer), in equal proportions. Such a ratio has been previously optimized to yield structures characterized by good structural integrity and low surface roughness. The organic ring in the SR368 monomer contributes to the final structure by promoting structural rigidity, while the ethoxylated groups in SR499, due to their long chains, help reduce mechanical shrinkage following photopolymerization.^37^ Additionally, SR399 imparts good mechanical strength to the structure as its pentaacrylate composition generates multiple polymer branches upon polymerization, thereby reinforcing the overall structure.^38^ These monomers have been employed in several biological studies, indicating biocompatibility with different microorganisms.^19,21,39,40^ Furthermore, Irgacure TPO-L (BASF) served as the photoinitiator, incorporated at a concentration of 3% relative to the total weight.

The microenvironments were characterized using scanning electron microscopy (SEM-HITACHI microscope, model TM3000). The fabricated microenvironments, specifically CRs and PGs were immersed in ethanol for 1 day to leach out the unpolymerized toxic monomer and then rinsed with distilled water. Finally, the samples were sterilized by UV-light irradiation for 1 h.

To study the growth of probiotics in microenvironments, a pool of bacteria with probiotic strains (Probiatop) was prepared, consisting of Lactobacillus acidophilus, Lactobacillus rhamnosus, Lactobacillus paracasei, and Bifidobacterium lactis.

For this purpose, glass slides containing the microstructures were immersed for 12, 18, 24, and 36 h in cultures containing the previously prepared probiotic strains (Probiatop) following the manufacturer’s preparation instructions (one sachet dissolved in 100 mL of tryptic soy broth culture medium, stirred with the aid of a spatula until completely homogenized) in a bacteriological incubator at 37 degrees.

The glass slides containing the microstructures exposed to the probiotic culture at different times were washed with 0.89% NaCl, and the samples were fixed using 2.5% glutaraldehyde solution (1 h at room temperature). Then, the samples were washed three times with 0.89% NaCl, dehydrated [by incubation in 70% ethanol (1×/1 h), 90% ethanol (1×/1 h) and 99% ethanol (5×/30 min)], and dried in a silica vacuum desiccator (7 days). Subsequently, images of the samples were acquired using SEM (HITACHI microscope, model TM3000).

## 3 Results and Discussion

Figure 1 shows the SEM images of the fabricated microenvironments composed of (a) CRs and (b) PGs; those are the microstructures used to study probiotics development. The CRs exhibit heights ranging from 4 to 10 μm, with an average thickness of 3.8 μm and an average spacing of approximately 4.4 μm, resulting in a total diameter of around 94 μm. On the other hand, the PGs have heights varying between 3 and 5 μm, with an average thickness and spacing of 4.5 μm and a total width of about 90 μm. Furthermore, the SEM micrograph reveals that the microenvironment exhibits good integrity and definition.

**Figure 1:**
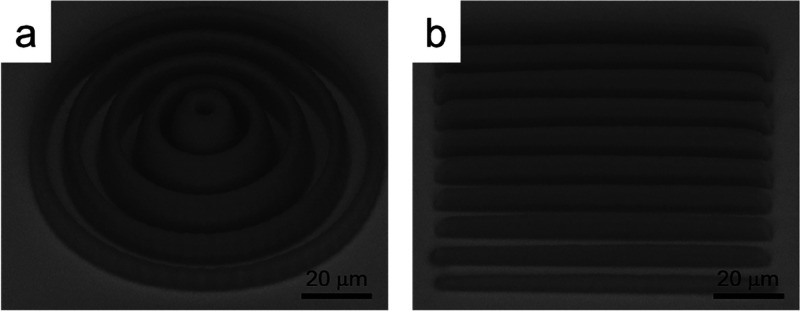
SEM micrographs of the microenvironment: (a) CRs and (b) PGs.

The microenvironments were inoculated (10^9^ bacteria/mL) and kept under all necessary conditions for their development. The bacterial growth was monitored at different times (12, 18, 24, and 36 h) by SEM.

Figure 2 shows the SEM image composed of sections of the CRs microenvironment for different incubation times. Bacterial growth in the region between rings of the structure, with some clusters, and significant bacterial adhesion to the walls of the structures are observed. It can be seen that there is an increase in the bacterial density with the incubation time, with a high prevalence of bacteria along the walls and grooves of the structures. In an analysis over 36 h, we also observed more bacteria adhesion on the top of the polymerized structures, as seen in Figure 2d. This result indicates a greater density of bacteria grown in the environment.

**Figure 2:**
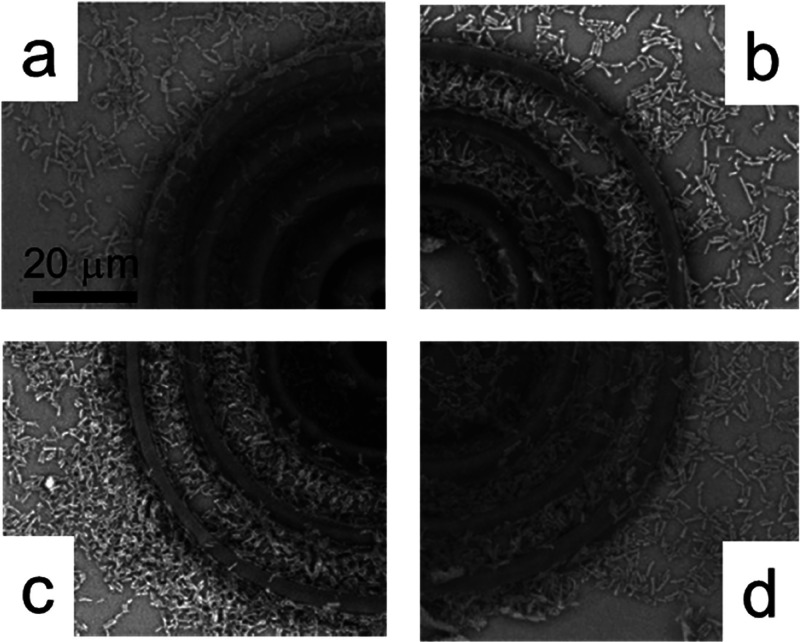
SEM micrographs of the CRs microenvironment inoculated with the probiotic pool after 12 h (a), 18 h (b), 24 h (c), and 36 h (d).

Similarly, the same microbial growth pattern is observed for the PG structures. As shown in Figure 3b, after 36 h of cultivation, there is a high prevalence of bacteria within the grooves between the structure walls. This indicates that bacteria prefer to proliferate in the protected environment between the parallel fabricated structures. This raises the hypothesis that certain regions within the microstructure may be more favorable for the development of the strain used compared to other *loci* due to factors such as intraspecific competition and spatial arrangement of the culture medium in relation to the geometry of the fabricated structure.

**Figure 3:**
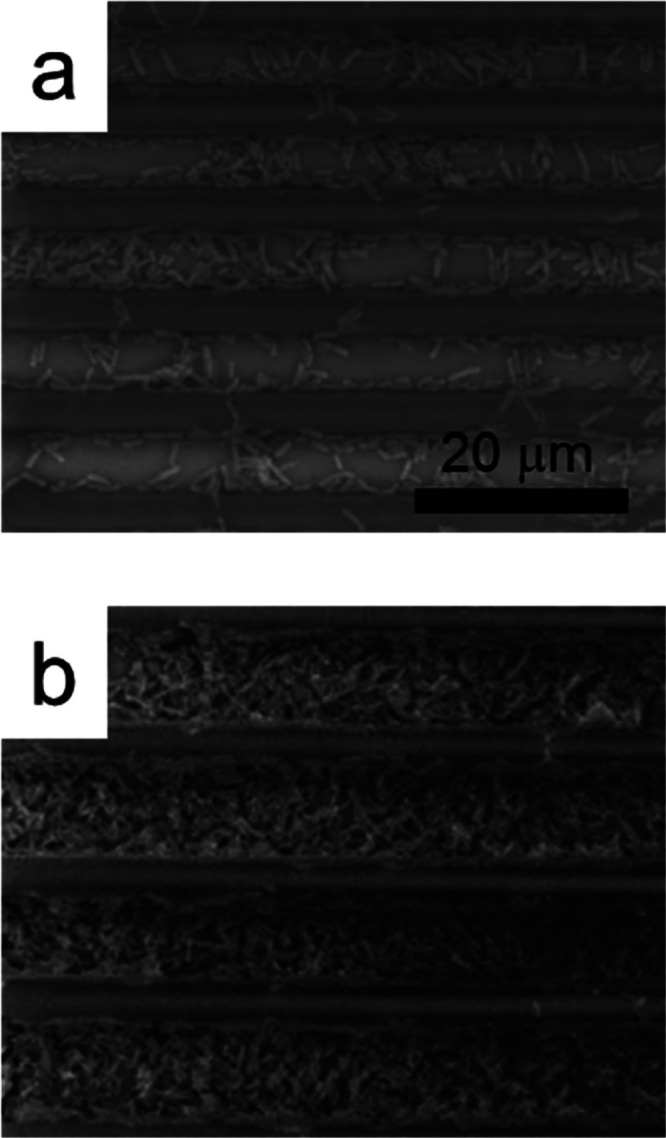
SEM micrographs of the PGs microenvironment inoculated with the probiotic pool after 12 (a) and 36 h (b).

By analyzing the SEM micrographs similar to the ones displayed in Figures 2 and 3, using ImageJ software, we were able to evaluate the percentage area occupied by the bacteria (*A*_b_) in the microenvironment and in a region far away from any structure (free space). Such a quantity is referred to as the bacterial density, ρ_bac_, in area %. It is important to mention that such a parameter refers to the cross-sectional area (as seen from the top). Figure 4 displays ρ_bac_ as a function of the incubation time for the CRs (red circles) and PGs (green circles) microenvironments. The blue circles in Figure 4 correspond to the bacterial density observed for a region without any microenvironment, i.e., in free space (FS). As it can be seen, at any given time, the growth of the probiotic (ρ_bac_) is higher in the microenvironments than in the FS. Comparing the microenvironments studied here, a higher bacterial density is observed for the CR structure. Furthermore, such results indicate a faster growth rate for the CRs microenvironment, followed by the PGs, with the smallest growth rate being observed in the FS condition. From the slopes of the linear fit presented in Figure 4, it was possible to determine the bacterial growth speed, v, given in area %/h. With such a parameter, we can quantify the distinct growth rate observed; from the FS to the PGs microenvironment, v increased from 0.28 to 0.63 area %/h, which corresponds to an increase of approximately 2-fold. However, a bacterial growth speed of 1.1 area %/h in the CRs environment was observed, corresponding to an increase of about 1.7. Such results clearly indicate that the microenvironments lead not only to a higher density of bacteria but also to a faster bacterial growth, therefore acting as accelerators to bacterial growth, reinforcing the observation made by the qualitative analysis of the SEM images described previously. The behavior observed in Figure 4 indicates that bacteria prefer to proliferate in the protected environment of fabricated structures; even the lateral opening in the PG structures poses a disadvantage for bacterial development with respect to the CRs microenvironment, as seen in the results of Figure 4. Hence, such results support the perception obtained from the SEM images (Figures 2 and 3) that excessively large zones are unfavorable for the full development of the probiotic pool.

**Figure 4:**
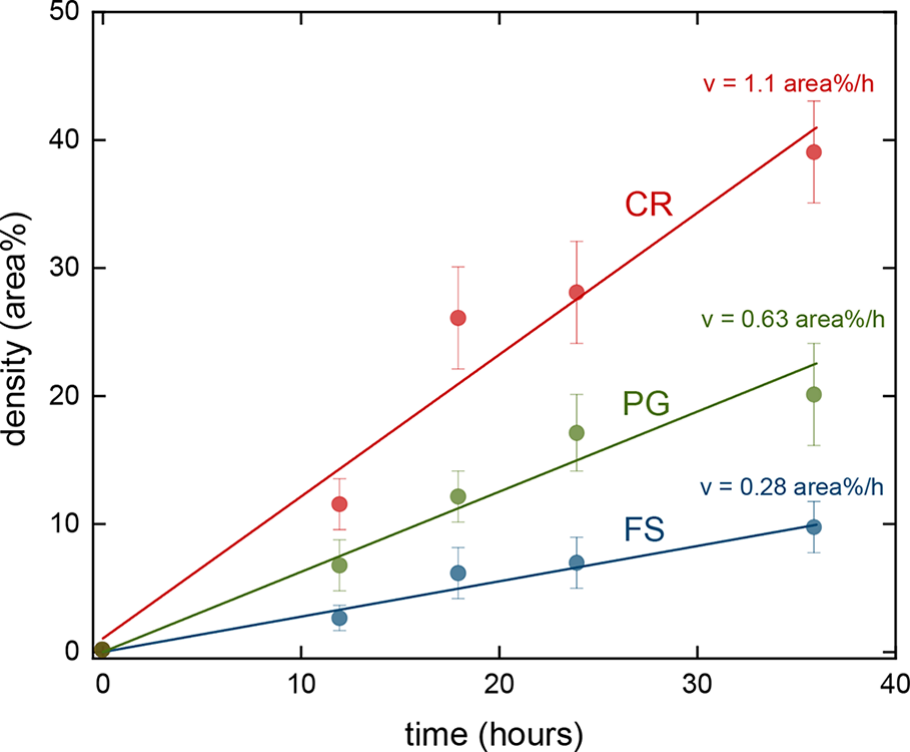
Density (in area %) of bacteria as a function of time for growth in different environments. The red line corresponds to the CRs microenvironment, while the green line corresponds to the PGs one. The growth in the FS is displayed as a blue line. The error bars show the standard deviations calculated from five individual analyses.

To further investigate the effect of geometrical aspects of microstructures on bacterial growth, based on the previous results, we explored the influence of the dimension of the zones on the development of probiotics by fabricating microstructures with different wall spacings. Figure 5 displays PGs microenvironments produced with varying distances between the walls (d; from ∼6 to ∼20 μm), inoculated with the probiotics pool for 24 h.

**Figure 5:**
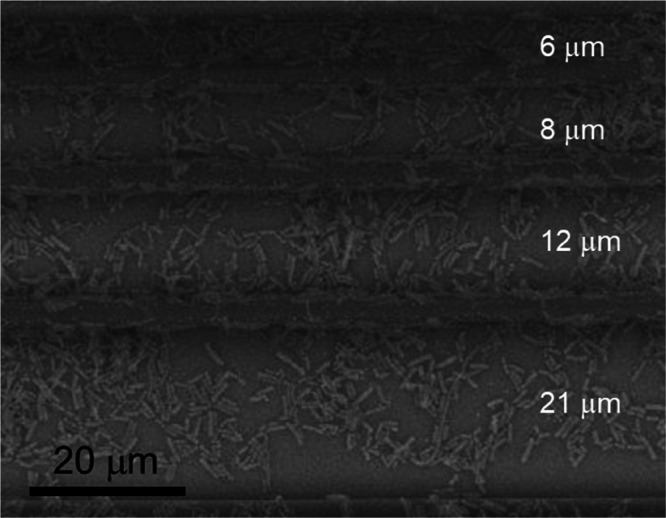
SEM micrograph of the PGs microenvironment with distinct spacing between the walls (noted in white) inoculated with the probiotic pool after 24 h of incubation.

Performing an analysis of the SEM images similar to the one shown in Figure 5, following the same procedure described before, we evaluated the bacterial density, ρ_bac_, in the microenvironment zones with a distinct spacing. Figure 6 shows ρ_bac_ as a function of the distance between the PG walls, d. As it can be seen, the bacterial density decreases by approximately a factor of 2 when the spacing in the microstructure increases from 5 to 17 μm, which can be interpreted as an indication that the presence of overly large areas in the structure may have contributed to inhibiting better bacterial development in comparison to the areas where the fabricated lines are closer together. Thus, extensive areas for bacterial cultivation are not the best promoters of bacterial cluster formation because the bacteria tend to spread across the entire surface before forming clusters. At the same time, it can be inferred that sites with very small dimensions are not particularly conducive to forming bacterial clusters because they can only support a limited number of microorganisms. From this, it can be inferred that an optimal groove size on the order of 5 μm favors the formation of bacterial clusters and potential biofilms.

**Figure 6:**
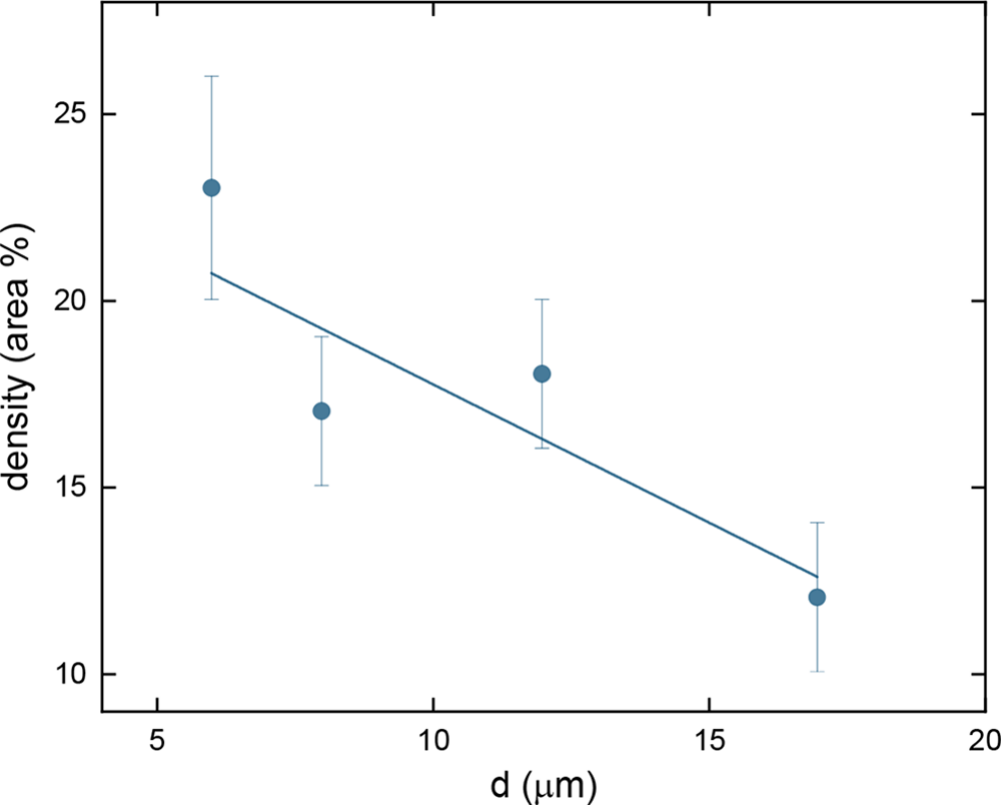
Density (in area %) of bacterial growth in PGs microenvironment with different distances between walls (Figure 5) after 24 h of incubation. The error bars show standard deviations calculated from five individual analyses.

## 4 Conclusions

In this work, we manufactured biocompatible environments with different geometries to evaluate the development of probiotic microorganisms (*Lactobacillus* and *Bifidobacterium* genera). Our results demonstrated that the microstructures are accelerators for bacterial growth, regardless of the employed geometry. Furthermore, it was observed that bacteria grow preferentially in protected environments with spacing on the order of 5 μm. The bacterial density decreases by approximately a factor of 2 when the spacing in the microstructure increases approximately 3 times. This may indicate that relatively large distances in the microenvironment may hinder bacterial growth compared with areas where the manufactured lines are closer. The presented study is a promising way to evaluate the biofilm formation from probiotic organisms cultivated in biomimetic environments, opening new possibilities for complex biomedical analyses.
